# Two-Dimensional DOA and Polarization Estimation for a Mixture of Uncorrelated and Coherent Sources with Sparsely-Distributed Vector Sensor Array

**DOI:** 10.3390/s16060789

**Published:** 2016-05-31

**Authors:** Weijian Si, Pinjiao Zhao, Zhiyu Qu

**Affiliations:** College of Information and Communication Engineering, Harbin Engineering University, Harbin 150001, China; swj0418@263.net (W.S.); zhaopinjiao@hrbeu.edu.cn (P.Z.)

**Keywords:** DOA estimation, polarization estimation, uncorrelated and coherent sources, sparsely-distributed vector sensor array

## Abstract

This paper presents an L-shaped sparsely-distributed vector sensor (SD-VS) array with four different antenna compositions. With the proposed SD-VS array, a novel two-dimensional (2-D) direction of arrival (DOA) and polarization estimation method is proposed to handle the scenario where uncorrelated and coherent sources coexist. The uncorrelated and coherent sources are separated based on the moduli of the eigenvalues. For the uncorrelated sources, coarse estimates are acquired by extracting the DOA information embedded in the steering vectors from estimated array response matrix of the uncorrelated sources, and they serve as coarse references to disambiguate fine estimates with cyclical ambiguity obtained from the spatial phase factors. For the coherent sources, four Hankel matrices are constructed, with which the coherent sources are resolved in a similar way as for the uncorrelated sources. The proposed SD-VS array requires only two collocated antennas for each vector sensor, thus the mutual coupling effects across the collocated antennas are reduced greatly. Moreover, the inter-sensor spacings are allowed beyond a half-wavelength, which results in an extended array aperture. Simulation results demonstrate the effectiveness and favorable performance of the proposed method.

## 1. Introduction

Direction of arrival (DOA) estimation using a sensor array has been a fundamental issue in many practical applications involving radar, wireless communication systems, and navigation [1,2]. The vector sensor array [3,4], which can make full use of the polarization diversity of the impinging sources, has shown significant superiority for DOA estimation as compared to the traditional scalar sensor array. Thus, the issue of DOA estimation with vector sensor array has attracted extensive attention during the last decades [5,6,7,8,9,10]. In the related studies, the polarized MUSIC-based methods [5,6,7] and the polarized ESPRIT-based methods [8,9,10] are two major kinds of approaches, and can achieve satisfactory performance in the case of uncorrelated sources. However, in practical environments, sources from an identical target may go through reflection from various surfaces, and hence the received sources may be a mixture of uncorrelated and coherent sources. In such environments, the methods mentioned above would suffer from serious performance deterioration owing to the rank deficiency of array covariance matrix caused by the multipath propagation.

To solve this problem, several decorrelation methods with vector sensor arrays have been investigated [11,12,13,14,15,16,17,18], where the DOAs are extracted on the basis of the polarization diversity and spatial diversity. References [11,12,13,14] are proposed using the spatially collocated six-component vector sensor arrays, and the key idea of these methods is to restore the rank of source subspace by averaging the data covariance matrices corresponding to each electric or magnetic component. Rahamim *et al.* [11] first developed a polarization smoothing (PS) technique to address the coherent sources using a vector sensor array. He *et al.* proposed a polarization difference smoothing (PDS) method [12] by incorporating the propagator method. Xu *et al.* presented a polarization angular smoothing (PAS) technique [13] by taking advantage of the cross-correlations among six subarrays. Subsequently, based on the rotational invariance, an improved polarization angular smoothing (IPAS) method was addressed in [14] to cope with the scenarios where uncorrelated and coherent sources coexist. However, the spatially collocated six-component vector sensor arrays used in these methods are easily subjected to the mutual coupling effects across the collocated antennas (hereinafter referred to as mutual coupling effects). Besides, the inter-sensor spacings of these arrays are required within a half-wavelength in accordance with the spatial Nyquist sampling theorem, thus the DOA estimation accuracy of these methods is limited to some extent [19,20,21]. Moreover, these methods fail to provide the estimation of polarization parameters. Since the mutual coupling effects have severe disturbances on the received sources, some efforts have been made to alleviate the mutual coupling effects [15,16]. A parallel factor analysis-based DOA-polarization estimation method [15] was investigated by using a tripole sensor array, where the collocated antennas of each vector sensor are reduced from six to three, and hence the mutual coupling effects are reduced. In [16], a DOA and polarization estimation method with a co-centered orthogonal loop and dipole (COLD) array was proposed by introducing the sparse Bayesian learning technique, and the mutual coupling effects are alleviated since the number of the collocated antennas of each vector sensor is reduced to two. On the other hand, to extend the array aperture, the sparsely-distributed vector sensor (SD-VS) array [10] with the inter-sensor spacings beyond a half-wavelength has been adopted [17,18]. Gu *et al.* [17] proposed a propagator-based DOA and polarization estimation method by exploiting the planar-plus-an-isolated sensor array, and the array aperture is extended owing to the inherent structure of the array. In [18], a well-separated dipole-loop sensor array was presented for expanding the array aperture, and DOA estimation accuracy was improved accordingly.

In view of the fact that the aforementioned “decorrelating” methods mainly focus on dealing with the coherent sources, while the coexistence of both uncorrelated and coherent sources is a common situation for many applications due to the multipath propagation [22,23]. It has been demonstrated that the PAS method can be extended to the scenario where uncorrelated and coherent sources coexist [14]. Unfortunately, it suffers from the mutual interference between uncorrelated and coherent sources and the low utilization of array aperture due to the simultaneous estimation of uncorrelated and coherent sources. Although the IPAS method specially considers the estimation for the DOA of uncorrelated and coherent sources by taking advantage of the spatial differencing theory, which however causes power loss of coherent sources. In summary, the existing solutions to the problem of DOA estimation under the coexistence of uncorrelated and coherent sources are confronted with two main difficulties: (1) how to separate the uncorrelated sources from coherent sources effectively; (2) how to reduce the mutual coupling effects and extend the array aperture simultaneously. In addition, considering the importance of polarization information for DOA resolution, and further for target classification and recognition, it is a critical issue to estimate the polarization parameters of mixed sources along with the DOA parameters.

To address these issues, we present an L-shaped sparsely-distributed vector sensor (SD-VS) array with four different antenna compositions in this paper, which provides two notable advantages:

(1). The mutual coupling effects are alleviated benefiting from the reduced collocated antennas of each vector sensor.
(2). The array aperture is extended by expanding the inter-sensor spacings beyond a half-wavelength.

With the proposed SD-VS array, a novel 2-D DOA and polarization estimation method for a mixture of uncorrelated and coherent sources is proposed. On the basis of the modulus property of the eigenvalues, the uncorrelated sources are firstly separated from the coherent sources, and hence the mutual interference between these two kinds of sources is avoided. For the uncorrelated sources, the coarse estimates are derived by exploiting the DOA information embedded in the polarization steering vectors which are obtained from the estimation of the uncorrelated array response matrix, and the fine estimates with cyclical ambiguity are obtained from spatial phase factors. In order to achieve the refined DOAs with no cyclical ambiguity, the coarse estimates are used for disambiguating the fine estimates. For the separated coherent sources, four Hankel matrices are constructed for the purpose of “decorrelating”, with which the coherent coarse estimates and the coherent fine estimates with cyclical ambiguity are calculated in a similar way as for the uncorrelated sources. Also, the coherent coarse estimates serve as references for the coherent fine estimates with cyclical ambiguity. Simulation results show the effectiveness and the improved estimate accuracy of the proposed method.

The mathematical notations used throughout this paper are denoted as follows. Vectors and matrices are denoted by lowercase and uppercase bold-face italic letters, respectively. ${\left(\cdot \right)}^{T}$, ${\left(\cdot \right)}^{*}$, ${\left(\cdot \right)}^{H}$, ${\left(\cdot \right)}^{-1}$, ${\left(\cdot \right)}^{†}$, ⊗, and E{⋅} denote transpose, conjugate, conjugate transpose, inverse, Moore-Penrose inverse, Kronecker product, and the statistical expectation, respectively. $0_{m\times n}$ is an null matrix and $I_{m}$ is an m×m identity matrix. ∠ denotes the angle of the ensuing entity, and |⋅| denotes the modulus of the internal entity. Additionally, det(⋅) and rank(⋅) are the determinant and the rank of the embraced matrix. diag{⋅} and blkdiag{⋅} denote a diagonal matrix and a block diagonal matrix, respectively. Re(⋅) and Im(⋅) are the real and the imaginary part of the embraced matrix. Furthermore, ⌊⋅⌋ and ⌈⋅⌉ are the floor and ceil operators.

The remainder of this paper is organized as follows. The proposed array configuration and source estimation model for mixed sources are given in Section 2. Section 3 presents the proposed DOA and polarization estimation method for a mixture of uncorrelated and coherent sources in detail. The computational complexity, several individual properties, and the extension of the proposed method are discussed in Section 4. Section 5 exhibits the simulation results of the proposed method. Conclusions are drawn in Section 6.

## 2. Array Configuration and Problem Formulation

### 2.1. Array Configuration Used in This Work

The six-component vector sensor array [11,12,13,14] is widely used for the estimation of DOA and polarization parameters. In general, each six-component vector sensor is composed of three orthogonally oriented dipoles plus three orthogonally oriented loops (spatially collocated in a point-like geometry), which is easily subjected to the mutual coupling effects. To reduce the mutual coupling effects, a new array configuration with four different antenna compositions is proposed as follows.

Consider an L-shaped SD-VS array consisting of dipole-dipole, loop-loop or dipole-loop antenna pairs distributed along the *x*-axis and *y*-axis with the inter-sensor spacings far larger than a half-wavelength (*i.e.*, Δx≫λ/2 and Δy≫λ/2, λ denotes the source wave length). For convenience, here we denote the dipole and the loop parallel to the *x*-axis as the *x*-dipole and *x*-loop, respectively, and the same is true for the *y*-dipole, *y*-loop, *z*-dipole, and *z*-loop. As demonstrated in [24], to make sure that the closed-form estimation-formulas are available, the number of linearly independent real-valued equations must be no less than the number of unknown parameters (elevation angle, azimuth angle, auxiliary polarization angle, and the polarization phase difference), which is referred to as the determined or over-determined conditions. Thus, the proposed array is configured with three constraint conditions:

(1). *x*-dipoles or *x*-loops must be placed on the *x*-axis, and *y*-dipoles or *y*-loops must be placed on the *y*-axis.
(2). If *x*-dipoles are placed on the *x*-axis, the corresponding *y*-dipoles are placed on the *y*-axis, and if *x*-loops are placed on the *x*-axis, the corresponding *y*-loops are placed on the *y*-axis.
(3). *z*-dipoles or *z*-loops must be placed on the *x*-axis and *y*-axis simultaneously.

**Figure 1:**
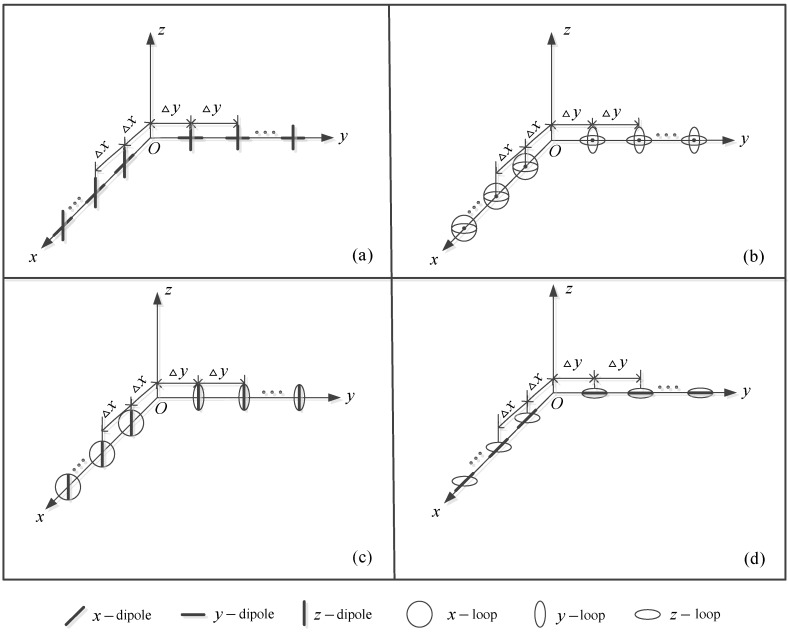
The four different antenna compositions of the L-shaped SD-VS array (**a**) dipole-dipole pairs; (**b**) loop-loop pairs; (**c**) dipole-loop pairs with *z*-dipoles; (**d**) loop-dipole pairs with *z*-loops.

According to the above constraint conditions, there exist four different antenna compositions in the SD-VS array, as depicted in Figure 1. Compared with the spatially collocated six-component vector sensor array, the proposed SD-VS array has the following two advantages:

(1). Since the proposed SD-VS array is composed of dipole-dipole, loop-loop, or dipole-loop antenna pairs, it only requires two collocated antennas for each vector sensor. Hence, the mutual coupling effects are alleviated greatly. Moreover, the antenna hardware costs are reduced.
(2). The inter-sensor spacings are allowed beyond a half-wavelength, which results in an extended array aperture, and the DOA estimation accuracy is improved accordingly.

Note that Wong [15,25] proposed six permutations of an electromagnetic vector sensor constituted by spatially noncollocating component-antennas which also have the same advantages (advantages 1 and 2 mentioned above) as the proposed SD-VS array. However, the DOA and polarization estimation method in [15,25] is developed based on a vector sensor, not a vector sensor array (*i.e.*, the maximum number of available antennas is six), thus the number of resolvable sources is limited.

### 2.2. Problem for Mulation and Modeling

Note that the following analysis is similar for all the four antenna compositions, we here take composition (a) as an example to derive the DOA and polarization estimation method for a mixture of uncorrelated and coherent sources. Consider K completely polarized narrow-band transverse electromagnetic (TEM) waves impinging on this array with $M\;(M=M_{1}+M_{2})$ vector sensors, where the number of dipoles or loops parallel to the *x*-axis, *y*-axis and *z*-axis are $M_{1}$, $M_{2}$ and $M_{1}+M_{2}$, respectively. The electric-field vector e measured by dipoles and the magnetic-field vector h measured by loops can be expressed as [15].

(1) $$c=\left[\begin{array}{l} e\\ h \end{array}\right]=\left[\begin{array}{l} e_{x}\\ e_{y}\\ e_{z}\\ h_{x}\\ h_{y}\\ h_{z} \end{array}\right]=\left[\begin{array}{c} \cos\phi \;\cos\theta \;\sin\gamma e^{j\eta }-\sin\phi \;\cos\gamma \\ \sin\phi \;\cos\theta \;\sin\gamma e^{j\eta }+\cos\phi \;\cos\gamma \\ -\sin\theta \;\sin\gamma e^{j\eta }\\ -\sin\phi \;\sin\gamma e^{j\eta }-\cos\phi \;\cos\theta \;\cos\gamma \\ \cos\phi \;\sin\gamma e^{j\eta }-\sin\phi \;\cos\theta \;\cos\gamma \\ \sin\theta \;\cos\gamma \end{array}\right]$$

where θ∈[0, π/2) signifies the elevation angle measured from the positive *z*-axis; ϕ∈[0, 2π) signifies the azimuth angle measured from the positive *x*-axis; γ∈[0, π/2) signifies the auxiliary polarization angle; and η∈[−π, π) signifies the polarization phase difference. Thus, the *x*-axis and *y*-axis polarization steering vectors are given by

(2) $$c_{x}=\left[\begin{array}{l} e_{x}\\ e_{z} \end{array}\right]=\left[\begin{array}{c} \cos\phi \;\cos\theta \;\sin\gamma e^{j\eta }-\sin\phi \;\cos\gamma \\ -\sin\theta \;\sin\gamma e^{j\eta } \end{array}\right]$$

(3) $$c_{y}=\left[\begin{array}{l} e_{y}\\ e_{z} \end{array}\right]=\left[\begin{array}{c} \sin\phi \;\cos\theta \;\sin\gamma e^{j\eta }+\cos\phi \;\cos\gamma \\ -\sin\theta \;\sin\gamma e^{j\eta } \end{array}\right]$$

The impinging sources, parameterized by $\left\{\theta_{1},\phi_{1},\gamma_{1},\eta_{1}\right\},\left\{\theta_{2},\phi_{2},\gamma_{2},\eta_{2}\right\},\cdots ,\left\{\theta_{K},\phi_{K},\gamma_{K},\eta_{K}\right\}$, are composed of $K_{u}$ uncorrelated sources and D groups (each group has $p_{k}$ coherent sources) of $K_{c}$ coherent sources, which satisfy $K_{c}=K-K_{u}=\sum_{k=1}^{D}p_{k}$. The entire 2M×1 array output vector of the proposed array at time t is written as

(4) $$x\left(t\right)=\sum_{k=1}^{K_{u}}a\left(\theta_{k},\phi_{k},\gamma_{k},\eta_{k}\right)s_{k}\left(t\right)+\sum_{k=K_{u}+1}^{K_{u}+D}\;\sum_{p=1}^{p_{k}}a\left(\theta_{k,p},\phi_{k,p},\gamma_{k,p},\eta_{k,p}\right)\varsigma_{k,p}s_{k}\left(t\right)+n\left(t\right)$$

where $a\left(\theta_{k},\phi_{k},\gamma_{k},\eta_{k}\right)$ is the 2M×1 steering vector of the entire SD-VS array, which is given by

(5) $$a_{k}={\left[{\left(Q_{x,k}⊗c_{x,k}\right)}^{T},{\left(Q_{y,k}⊗c_{y,k}\right)}^{T}\right]}^{T}$$

where $c_{x,k}$ and $c_{y,k}$ are given by Equations (2) and (3), respectively, with respect to the *k*th impinging source. $Q_{x,k}={\left[q_{x,k},q_{x,k}^{2},\cdots ,q_{x,k}^{M_{1}}\right]}^{T}$ with $q_{x,k}=e^{j2\pi u_{k}\Delta x/\lambda }$ and $u_{k}=\sin\theta_{k}\cos\phi_{k}$ being the spatial phase factor and the direction-cosine along the *x*-axis, and $Q_{y,k}={\left[q_{y,k},q_{y,k}^{2},\cdots ,q_{y,k}^{M_{2}}\right]}^{T}$ with $q_{y,k}=e^{j2\pi v_{k}\Delta y/\lambda }$ and $v_{k}=\sin\theta_{k}\sin\phi_{k}$ being the spatial phase factor and the direction-cosine along the *y*-axis.

Equation (4) can be further rewritten as

(6) $$\begin{array}{ll} x\left(t\right) & =A_{u}\left(\theta ,\phi ,\gamma ,\eta \right)s_{u}\left(t\right)+A_{c}\left(\theta ,\phi ,\gamma ,\eta \right)\Gamma s_{c}\left(t\right)+n\left(t\right)\\ & =A\left(\theta ,\phi ,\gamma ,\eta \right)Es\left(t\right)+n\left(t\right) \end{array}$$

where $s\left(t\right)={\left[s_{1}\left(t\right),s_{2}\left(t\right),\cdots ,s_{K}\left(t\right)\right]}^{T}$ and n(t) are the source and noise vectors, respectively. For brevity, we define $s\left(t\right)={\left[s_{u}^{T}\left(t\right),s_{c}^{T}\left(t\right)\right]}^{T}$ with $s_{u}\left(t\right)={\left[s_{1}\left(t\right),s_{2}\left(t\right),\cdots ,s_{K_{u}}\left(t\right)\right]}^{T}$ and $s_{c}\left(t\right)={\left[s_{K_{u}+1}\left(t\right),s_{K_{u}+2}\left(t\right),\cdots ,s_{K_{u}+D}\left(t\right)\right]}^{T}$ as being the source vectors associated with the uncorrelated and coherent sources, respectively. $E=\text{blkdiag}\left\{I_{K_{u}},\Gamma \right\}$ is a $K\times (K_{u}+D)$ block diagonal matrix, and Γ is the fading coefficient matrix whose the *k*th column is expressed as $\varsigma_{k}={\left[\varsigma_{k,1},\varsigma_{k,2},\cdots ,\varsigma_{k,p_{k}}\right]}^{T}$ for $k=K_{u}+1,K_{u}+2,\cdots ,K_{u}+D$. $A\left(\theta ,\phi ,\gamma ,\eta \right)=\left[A_{u}\left(\theta ,\phi ,\gamma ,\eta \right),A_{c}\left(\theta ,\phi ,\gamma ,\eta \right)\right]$ is the array response matrix of size 2M×K, in which $A_{u}\left(\theta ,\phi ,\gamma ,\eta \right)=[a\left(\theta_{1},\phi_{1},\gamma_{1},\eta_{1}\right),$ $a\left(\theta_{2},\phi_{2},\gamma_{2},\eta_{2}\right),\cdots ,a\left(\theta_{K_{u}},\phi_{K_{u}},\gamma_{K_{u}},\eta_{K_{u}}\right)]$ and $A_{c}\left(\theta ,\phi ,\gamma ,\eta \right)=[A_{c,K_{u}+1}\left(\theta ,\phi ,\gamma ,\eta \right),A_{c,K_{u}+2}\left(\theta ,\phi ,\gamma ,\eta \right),\cdots ,$ $A_{c,K_{u}+D}\left(\theta ,\phi ,\gamma ,\eta \right)]$ are the array manifold matrices corresponding to $K_{u}$ uncorrelated sources and $K_{c}$ coherent sources respectively with $A_{c,k}\left(\theta ,\phi ,\gamma ,\eta \right)=[a\left(\theta_{k,1},\phi_{k,1},\gamma_{k,1},\eta_{k,1}\right),a\left(\theta_{k,2},\phi_{k,2},\gamma_{k,2},\eta_{k,2}\right),\cdots ,a\left(\theta_{k,p_{k}},\phi_{k,p_{k}},\gamma_{k,p_{k}},\eta_{k,p_{k}}\right)]$ being the array response matrix of the *k*th coherent group. The array output of N snapshots collected by the SD-VS array can be represented by

(7) $$\begin{array}{ll} X & =A_{u}\left(\theta ,\phi ,\gamma ,\eta \right)S_{u}+A_{c}\left(\theta ,\phi ,\gamma ,\eta \right)\Gamma S_{c}+N\\ & =A\left(\theta ,\phi ,\gamma ,\eta \right)ES+N \end{array}$$

where X=[x(1),x(2),⋯,x(N)], $S_{u}=\left[s_{u}(1),s_{u}(2),\cdots ,s_{u}(N)\right]$, $S_{c}=\left[s_{c}(1),s_{c}(2),\cdots ,s_{c}(N)\right]$ and S=[s(1),s(2),⋯,s(N)]. The objective of the proposed method is to determine the 2-D DOA and polarization parameters $\left\{\theta_{k},\phi_{k},\gamma_{k},\eta_{k}\right\}$ k=1,2,⋯,K for a mixture of uncorrelated and coherent sources. For notational convenience, A(θ,ϕ,γ,η), $A_{u}\left(\theta ,\phi ,\gamma ,\eta \right)$, and $A_{c}\left(\theta ,\phi ,\gamma ,\eta \right)$ are respectively abbreviated as A, $A_{u}$, and $A_{c}$ in the following analysis.

The basic assumptions utilized throughout this paper are listed as follows.

(1). s(t) and n(t) are the two mutually uncorrelated zero-mean stationary Gaussian random processes.
(2). Coherent sources ${\left\{s_{k}(t)\right\}}_{k=K_{u}+1}^{K_{u}+D}$ from different coherent groups are uncorrelated with each other, and they are uncorrelated with the uncorrelated sources ${\left\{s_{k}(t)\right\}}_{k=1}^{K_{u}}$ as well.
(3). The number of uncorrelated sources, coherent sources, coherent groups and fading coefficients (*i.e.*, the values of $K_{u}$, $K_{c}$, D, $\varsigma_{k,p}$) can be estimated using the source number estimation method [26] and the fading coefficients estimation method [27].

## 3. 2-D Parameter Estimation

In this section, a 2-D DOA and polarization estimation method is proposed for a mixture of uncorrelated and coherent sources by using composition (a) of Figure 1 of the proposed SD-VS array.

### 3.1. Distinguish Uncorrelated Sources from Coherent Sources

The covariance matrix of X is written as

(8) $$\begin{array}{ll} R & =E\left\{XX^{H}\right\}=AER_{s}E^{H}A^{H}+\sigma_{n}^{2}I\\ & =A_{u}R_{u}A_{u}^{H}+A_{c}\Gamma R_{c}\Gamma^{H}A_{c}^{H}+\sigma_{n}^{2}I \end{array}$$

where $\sigma_{n}^{2}$ denotes the noise variance. $R_{s}=E\left\{SS^{H}\right\}$ denotes the source covariance matrix, $R_{u}=E\left\{S_{u}S_{u}^{H}\right\}$ and $R_{c}=E\left\{S_{c}S_{c}^{H}\right\}$ are the source covariance matrices related to the uncorrelated and the coherent sources, respectively. Due to the fact that the K impinging sources are composed of $K_{u}$ uncorrelated sources and D groups of $K_{c}$ coherent sources, $R_{s}$ is of rank $K_{u}+D$.

By performing eigenvalue decomposition (EVD) on R, $K_{u}+D$ larger eigenvalues are selected. Additionally, the source subspace $E_{s}$ can be constructed from the corresponding $K_{u}+D$ eigenvectors. It is well known that the columns of $E_{s}$ and AE span the same subspace, hence there must exist a unique full-rank matrix T which satisfies

(9) $$E_{s}=AET=\left[A_{u},A_{c}\Gamma \right]T=\left[\begin{array}{cc} C_{u}^{\left[x,z\right]}\Delta_{u_{x}} & C_{c}^{\left[x,z\right]}\Delta_{c_{x}}\Gamma \\ \vdots & \vdots \\ C_{u}^{\left[x,z\right]}\Delta_{u_{x}}^{M_{1}} & C_{c}^{\left[x,z\right]}\Delta_{c_{x}}^{M_{1}}\Gamma \\ C_{u}^{\left[y,z\right]}\Delta_{u_{y}} & C_{c}^{\left[y,z\right]}\Delta_{c_{y}}\Gamma \\ \vdots & \vdots \\ C_{u}^{\left[y,z\right]}\Delta_{u_{y}}^{M_{2}} & C_{c}^{\left[y,z\right]}\Delta_{c_{y}}^{M_{2}}\Gamma \end{array}\right]T$$

where $\Delta_{u_{x}}=\text{diag}\left\{q_{x,1},q_{x,2},\cdots ,q_{x,K_{u}}\right\}$ and $\Delta_{u_{y}}=\text{diag}\left\{q_{y,1},q_{y,2},\cdots ,q_{y,K_{u}}\right\}$ are two $K_{u}\times K_{u}$ diagonal matrices constituted by the *x*-axis and *y*-axis spatial phase factors of uncorrelated sources, and $\Delta_{c_{x}}=\text{diag}\{q_{x,K_{u}+1,1},\cdots ,q_{x,K_{u}+1,p_{1}},\cdots ,q_{x,K_{u}+D,1},\cdots ,q_{x,K_{u}+D,p_{D}}\}$ and $\Delta_{c_{y}}=\text{diag}\{q_{y,K_{u}+1,1},\cdots ,q_{y,K_{u}+1,p_{1}},\cdots ,$ $q_{y,K_{u}+D,1},\cdots ,q_{y,K_{u}+D,p_{D}}\}$ are two $K_{c}\times K_{c}$ diagonal matrices constituted by the *x*-axis and *y*-axis spatial phase factors of coherent sources. $C_{u}^{\left[x,z\right]}=\left[c_{x,1},c_{x,2},\cdots ,c_{x,K_{u}}\right]$, $C_{u}^{\left[y,z\right]}=\left[c_{y,1},c_{y,2},\cdots ,c_{y,K_{u}}\right]$, $C_{c}^{\left[x,z\right]}=[c_{x,K_{u}+1,1},\cdots ,c_{x,K_{u}+1,p_{1}},\cdots ,c_{x,K_{u}+D,1},\cdots ,c_{x,K_{u}+D,p_{D}}]$ and $C_{c}^{\left[y,z\right]}=\;[c_{y,K_{u}+1,1},\cdots ,c_{y,K_{u}+1,p_{1}},\cdots ,c_{y,K_{u}+D,1},$ $\cdots ,c_{y,K_{u}+D,p_{D}}]$.

According to the array configuration of the proposed SD-VS array, $E_{s}$ can be divided into four submatrices with the identical size, which is given by

(10) $$E_{s}={\left[{\left(E_{s}^{\left[x\right]}\right)}^{T},{\left(E_{s}^{\left[z_{x}\right]}\right)}^{T},{\left(E_{s}^{\left[y\right]}\right)}^{T},{\left(E_{s}^{\left[z_{y}\right]}\right)}^{T}\right]}^{T}$$

where

(11) $$E_{s}^{\left[x\right]}=G_{2M_{1},1}^{T}E_{s}^{\left[x,z\right]}$$

(12) $$E_{s}^{\left[z_{x}\right]}=G_{2M_{1},2}^{T}E_{s}^{\left[x,z\right]}$$

(13) $$E_{s}^{\left[y\right]}=G_{2M_{2},1}^{T}E_{s}^{\left[y,z\right]}$$

(14) $$E_{s}^{\left[z_{y}\right]}=G_{2M_{2},2}^{T}E_{s}^{\left[y,z\right]}$$

with $E_{s}^{\left[x,z\right]}$ and $E_{s}^{\left[y,z\right]}$ being the first $2M_{1}$ and the last $2M_{2}$ rows of $E_{s}$, and $G_{l,n}$ is an exchange matrix defined as

(15) $$G_{l,n}=\left[g_{n},g_{n+2},\cdots ,g_{n+l-2}\right],\;n=1,2$$

where $g_{i}$ is a l×1 unit vector with one on the *i*th row and zeros elsewhere. Intuitively, $E_{s}^{\left[x\right]}$, $E_{s}^{\left[z_{x}\right]}$, $E_{s}^{\left[y\right]}$ and $E_{s}^{\left[z_{y}\right]}$ are characteristic of inherent rotational-invariant structure, thus any one of them can be used for distinguishing uncorrelated sources from coherent sources. Here, we take $E_{s}^{\left[x\right]}$ as an example.

By taking advantage of the rotational invariance, $E_{s}^{\left[x\right]}$ can be divided into two overlapped submatrices, as

(16) $$E_{s}^{{\left[x\right]}_{1}}=J_{M_{1},1}E_{s}^{\left[x\right]}$$

(17) $$E_{s}^{{\left[x\right]}_{2}}=J_{M_{1},2}E_{s}^{\left[x\right]}$$

with the selection matrix $J_{l,n}=\left[0_{\left(l-1)\times (n-1\right)}\;I_{\left(l-1\right)}\;0_{\left(l-1)\times (2-n\right)}\right]$. Combining Equations (16) with (17) yields

(18) $${\left(E_{s}^{{\left[x\right]}_{1}}\right)}^{†}E_{s}^{{\left[x\right]}_{2}}=T^{-1}\Delta_{x}T$$

where $\Delta_{x}=\text{blkdiag}\left\{\Delta_{u_{x}},\Gamma^{†}\Delta_{c_{x}}\Gamma \right\}$ is a block diagonal matrix that contains the DOA information of both uncorrelated and coherent sources. As can be seen from Equation (18), $\Delta_{x}$ is constructed by extracting the $K_{u}+D$ larger eigenvalues of ${\left(E_{s}^{{\left[x\right]}_{1}}\right)}^{†}E_{s}^{{\left[x\right]}_{2}}$ and the full-rank matrix $T^{-1}$ is obtained from the corresponding $K_{u}+D$ eigenvectors. Based on modulus property outlined in [28], the moduli of the elements in $\Delta_{u_{x}}$ are approximately equivalent to 1 in the case of noise disturbance, *i.e.*, $\left|\left|\det\left(\Delta_{u_{x}}\right)\right|-1\right|=\varepsilon$ with ε→0 (in the noise-free case, ε=0), while those in $\Delta_{c_{x}}$ are far away from 1. Following this principle, the uncorrelated sources can be distinguished from the coherent sources.

In order to construct the uncorrelated eigenvector matrix $T_{u}^{-1}$, $K_{u}$ column vectors corresponding to uncorrelated sources are extracted from $T^{-1}$. Similarly, the remaining D column vectors corresponding to coherent sources are extracted to construct the matrix $T_{c}^{-1}$.

### 3.2. 2-D Parameter Estimation for Uncorrelated Sources

The estimation of $A_{u}$ is given by

(19) $${\hat{A}}_{u}=E_{s}T_{u}^{-1}=\left[\begin{array}{c} C_{u}^{\left[x,z\right]}\Delta_{u_{x}}\\ \vdots \\ C_{u}^{\left[x,z\right]}\Delta_{u_{x}}^{M_{1}}\\ C_{u}^{\left[y,z\right]}\Delta_{u_{y}}\\ \vdots \\ C_{u}^{\left[y,z\right]}\Delta_{u_{y}}^{M_{2}} \end{array}\right]$$

For the *k*th uncorrelated source, we have ${\hat{A}}_{u,k}=E_{s}T_{u,k}^{-1}$, where $T_{u,k}^{-1}$ is the eigenvector of the *k*th uncorrelated source selected from $T_{u}^{-1}$. With the definition of the exchange matrix $G_{l,n}$, ${\hat{A}}_{u,k}$ can be partitioned as

(20) $${\hat{A}}_{u,k}={\left[{\left({\hat{A}}_{u,k}^{\left[x\right]}\right)}^{T},{\left({\hat{A}}_{u,k}^{\left[z_{x}\right]}\right)}^{T},{\left({\hat{A}}_{u,k}^{\left[y\right]}\right)}^{T},{\left({\hat{A}}_{u,k}^{\left[z_{y}\right]}\right)}^{T}\right]}^{T}$$

where

(21) $${\hat{A}}_{u,k}^{\left[x\right]}=G_{2M_{1},1}^{T}{\hat{A}}_{u,k}^{\left[x,z\right]}$$

(22) $${\hat{A}}_{u,k}^{\left[z_{x}\right]}=G_{2M_{1},2}^{T}{\hat{A}}_{u,k}^{\left[x,z\right]}$$

(23) $${\hat{A}}_{u,k}^{\left[y\right]}=G_{2M_{2},1}^{T}{\hat{A}}_{u,k}^{\left[y,z\right]}$$

(24) $${\hat{A}}_{u,k}^{\left[z_{y}\right]}=G_{2M_{2},2}^{T}{\hat{A}}_{u,k}^{\left[y,z\right]}$$

with ${\hat{A}}_{u,k}^{\left[x,z\right]}$ and ${\hat{A}}_{u,k}^{\left[y,z\right]}$ the first $2M_{1}$ and the last $2M_{2}$ rows of ${\hat{A}}_{u,k}$.

Combining Equations (21) with (22) yields

(25) $$\frac{{\hat{e}}_{u,k}^{\left[x\right]}}{{\hat{e}}_{u,k}^{\left[z\right]}}={\left({\hat{A}}_{u,k}^{\left[z_{x}\right]}\right)}^{†}{\hat{A}}_{u,k}^{\left[x\right]}=\left[-\cot\theta_{k}\cos\phi_{k}+\cot\gamma_{k}\frac{\sin\phi_{k}}{\sin\theta_{k}}\cos\eta_{k}\right]+j\left[-\cot\gamma_{k}\frac{\sin\phi_{k}}{\sin\theta_{k}}\sin\eta_{k}\right]$$

In a similar way, by exploiting Equations (23) and (24), we have

(26) $$\frac{{\hat{e}}_{u,k}^{\left[y\right]}}{{\hat{e}}_{u,k}^{\left[z\right]}}={\left(\;{\hat{A}}_{u,k}^{\left[z_{y}\right]}\right)}^{†}{\hat{A}}_{u,k}^{\left[y\right]}=\left[-\cot\theta_{k}\sin\phi_{k}-\cot\gamma_{k}\frac{\cos\phi_{k}}{\sin\theta_{k}}\cos\eta_{k}\right]+j\left[\cot\gamma_{k}\frac{\cos\phi_{k}}{\sin\theta_{k}}\sin\eta_{k}\right]$$

According to Equations (25) and (26), we can obtain four real-valued equations: $\text{Re}\left({\hat{e}}_{u,k}^{\left[x\right]}/{\hat{e}}_{u,k}^{\left[z\right]}\right)=-\cot\theta_{k}\cos\phi_{k}+\cot\gamma_{k}\frac{\sin\phi_{k}}{\sin\theta_{k}}\cos\eta_{k}$, $\text{Im}\left({\hat{e}}_{u,k}^{\left[x\right]}/{\hat{e}}_{u,k}^{\left[z\right]}\right)=-\cot\gamma_{k}\frac{\sin\phi_{k}}{\sin\theta_{k}}\sin\eta_{k}$, $\text{Re}\left({\hat{e}}_{u,k}^{\left[y\right]}/{\hat{e}}_{u,k}^{\left[z\right]}\right)=-\cot\theta_{k}\sin\phi_{k}-\cot\gamma_{k}\frac{\cos\phi_{k}}{\sin\theta_{k}}\cos\eta_{k}$, $\text{Im}\left({\hat{e}}_{u,k}^{\left[y\right]}/{\hat{e}}_{u,k}^{\left[z\right]}\right)=\cot\gamma_{k}\frac{\cos\phi_{k}}{\sin\theta_{k}}\sin\eta_{k}$. Then, the closed-form estimation-formulas (*i.e.*, coarse estimates) of azimuth angle, elevation angle, auxiliary polarization angle, and the polarization phase difference are given by

(27) $${\hat{\phi }}_{k}^{coarse}=\left\{ \begin{array}{l} \tan^{-1}\left\{\frac{-\text{Im}\left({\hat{e}}_{u,k}^{\left[x\right]}/{\hat{e}}_{u,k}^{\left[z\right]}\right)}{\text{Im}\left({\hat{e}}_{u,k}^{\left[y\right]}/{\hat{e}}_{u,k}^{\left[z\right]}\right)}\right\},\;\mathrm{if}\;\left(\sin\eta_{k}\cdot \text{Im}\left({\hat{e}}_{u,k}^{\left[y\right]}/{\hat{e}}_{u,k}^{\left[z\right]}\right)\geq 0\right)\\ \tan^{-1}\left\{\frac{-\text{Im}\left({\hat{e}}_{u,k}^{\left[x\right]}/{\hat{e}}_{u,k}^{\left[z\right]}\right)}{\text{Im}\left({\hat{e}}_{u,k}^{\left[y\right]}/{\hat{e}}_{u,k}^{\left[z\right]}\right)}\right\}+\pi ,\;\mathrm{if}\;\left(\sin\eta_{k}\cdot \text{Im}\left({\hat{e}}_{u,k}^{\left[y\right]}/{\hat{e}}_{u,k}^{\left[z\right]}\right)<0\right)\; \end{array}\right.$$

(28) $$\begin{array}{l} {\hat{\theta }}_{k}^{coarse}=\left\{ \begin{array}{l} \tan^{-1}\left\{\frac{1}{H}\right\},\;\mathrm{if}\;\left(H\geq 0\right)\\ \tan^{-1}\left\{\frac{1}{H}\right\}+\pi ,\;\mathrm{if}\;\left(H<0\right)\; \end{array}\right.\\ H=-\text{Re}\left({\hat{e}}_{u,k}^{\left[x\right]}/{\hat{e}}_{u,k}^{\left[z\right]}\right)\cos{\hat{\phi }}_{k}^{coarse}-\text{Re}\left({\hat{e}}_{u,k}^{\left[y\right]}/{\hat{e}}_{u,k}^{\left[z\right]}\right)\sin{\hat{\phi }}_{k}^{coarse} \end{array}$$

(29) $${\hat{\eta }}_{k}^{coarse}=-∠\left(\left({\hat{e}}_{u,k}^{\left[x\right]}/{\hat{e}}_{u,k}^{\left[z\right]}\right)\sin{\hat{\phi }}_{k}^{coarse}-\left({\hat{e}}_{u,k}^{\left[y\right]}/{\hat{e}}_{u,k}^{\left[z\right]}\right)\cos{\hat{\phi }}_{k}^{coarse}\right)$$

(30) $${\hat{\gamma }}_{k}^{coarse}=\cot^{-1}\left(\frac{\text{Im}\left({\hat{e}}_{u,k}^{\left[y\right]}/{\hat{e}}_{u,k}^{\left[z\right]}\right)\sin{\hat{\phi }}_{k}^{coarse}}{\cos{\hat{\phi }}_{k}^{coarse}\sin{\hat{\eta }}_{k}^{coarse}}\right)$$

Based on the above derivation, the coarse estimates of composition (a) are achieved. Note that the composition (b) is made up of loop-loop pairs, thus Equations (2) and (3) are respectively replaced by $c_{x}={\left[h_{x}^{T},h_{z}^{T}\right]}^{T}$ and $c_{y}={\left[h_{y}^{T},h_{z}^{T}\right]}^{T}$. Similarly, for composition (c), we have $c_{x}={\left[h_{x}^{T},e_{z}^{T}\right]}^{T}$ and $c_{y}={\left[h_{y}^{T},e_{z}^{T}\right]}^{T}$; for composition (d), we have $c_{x}={\left[e_{x}^{T},h_{z}^{T}\right]}^{T}$ and $c_{y}={\left[e_{y}^{T},h_{z}^{T}\right]}^{T}$. As a result, the coarse estimates of the other three antenna compositions (composition (b), (c) and (d)) are obtained in the same manner as those of composition (a), which are given in Table 1. Due to the fact that these coarse estimates are irrespective of the sizes of the inter-sensor spacings Δx and Δy, they are unambiguous but inaccurate. Note that the method in [29] also derives the closed-form polarization-estimation formulas, but it operates on the premise that the DOA of the incident source is already known. By contrast, our approach can provide the closed-form estimation formulas for both DOA and polarization parameters. Additionally, our approach can provide the fine DOA estimates, which is given in the following description.

To obtain the fine estimates, we need to estimate the spatial phase factors ${\hat{q}}_{x,k}$ and ${\hat{q}}_{y,k}$ related to the array geometric aperture. By taking advantage of the inherent rotational-invariant structure of ${\hat{A}}_{u,k}^{\left[x\right]}$ and ${\hat{A}}_{u,k}^{\left[y\right]}$, we have

(31) $${\hat{q}}_{x,k}={\left({\hat{A}}_{u,k}^{{\left[x\right]}_{1}}\right)}^{†}{\hat{A}}_{u,k}^{{\left[x\right]}_{2}}$$

(32) $${\hat{q}}_{y,k}={\left({\hat{A}}_{u,k}^{{\left[y\right]}_{1}}\right)}^{†}{\hat{A}}_{u,k}^{{\left[y\right]}_{2}}$$

where

(33) $${\hat{A}}_{u,k}^{{\left[x\right]}_{1}}=J_{M_{1},1}{\hat{A}}_{u,k}^{\left[x\right]}$$

(34) $${\hat{A}}_{u,k}^{{\left[x\right]}_{2}}=J_{M_{1},2}{\hat{A}}_{u,k}^{\left[x\right]}$$

(35) $${\hat{A}}_{u,k}^{{\left[y\right]}_{1}}=J_{M_{2},1}{\hat{A}}_{u,k}^{\left[y\right]}$$

(36) $${\hat{A}}_{u,k}^{{\left[y\right]}_{2}}=J_{M_{2},2}{\hat{A}}_{u,k}^{\left[y\right]}$$

And the fine estimates of *x*-axis and *y*-axis direction-cosines are given by

(37) $$u_{u,k}^{fine}=\frac{\lambda }{2\pi \Delta x}∠{\hat{q}}_{x,k}$$

(38) $$v_{u,k}^{fine}=\frac{\lambda }{2\pi \Delta y}∠{\hat{q}}_{y,k}$$

Since the inter-sensor spacings Δx and Δy are beyond λ/2, cyclical ambiguity may exist in Equations (37) and (38), that is

(39) $${\hat{u}}_{u,k}=u_{u,k}^{fine}+m_{k}^{\circ }\frac{\lambda }{\Delta x}$$

(40) $${\hat{v}}_{u,k}=v_{u,k}^{fine}+n_{k}^{\circ }\frac{\lambda }{\Delta y}$$

where

(41) $$m_{k}^{\circ }=\underset{m_{k}}{\text{arg}\;\text{min}}\left|u_{u,k}^{coarse}-u_{u,k}^{fine}-\frac{m_{k}^{\circ }\lambda }{\Delta x}\right|$$

(42) $$n_{k}^{\circ }=\underset{n_{k}}{\text{arg}\;\text{min}}\left|v_{u,k}^{coarse}-v_{u,k}^{fine}-\frac{n_{k}^{\circ }\lambda }{\Delta y}\right|$$

for

(43) $$m_{k}^{\circ }\in \left\{\left\lceil \Delta x/\lambda \left(-1-u_{u,k}^{coarse}\right)\right\rceil ,\left\lfloor \Delta x/\lambda \left(1-u_{u,k}^{coarse}\right)\right\rfloor \right\}$$

(44) $$n_{k}^{\circ }\in \left\{\left\lceil \Delta y/\lambda \left(-1-v_{u,k}^{coarse}\right)\right\rceil ,\left\lfloor \Delta y/\lambda \left(1-v_{u,k}^{coarse}\right)\right\rfloor \right\}$$

where the coarse estimates of the *x*-axis and *y*-axis direction-cosines are defined by $u_{u,k}^{coarse}=\sin{\hat{\theta }}_{k}^{coarse}\cos{\hat{\phi }}_{k}^{coarse}$ and $v_{u,k}^{coarse}=\sin{\hat{\theta }}_{k}^{coarse}\sin{\hat{\phi }}_{k}^{coarse}$, which are used for disambiguation. As a result, the 2-D refined and unambiguous angles are given by ${\hat{\theta }}_{u,k}=\sin^{-1}\left(\sqrt{{\left({\hat{u}}_{u,k}\right)}^{2}+{\left({\hat{v}}_{u,k}\right)}^{2}}\right)$ and ${\hat{\phi }}_{u,k}=∠\left({\hat{u}}_{u,k}+j{\hat{v}}_{u,k}\right)$.

### 3.3. 2-D Parameter Estimation for Coherent Sources

Similar to the 2-D DOA and polarization estimation for the uncorrelated sources, the coherent sources are also resolved by three steps: (1) the coarse estimates; (2) the fine estimates with cyclical ambiguity; (3) using the coarse estimates to disambiguate the fine estimates.

The estimation of array response matrix with respect to the *k*th $\left(k=K_{u}+1,K_{u}+2,\cdots ,K_{u}+D\right)$ coherent group ${\hat{A}}_{c,k}$ is given by

(45) $${\hat{A}}_{c,k}=E_{s}T_{c,k}^{-1}=\left[\begin{array}{c} C_{c,k}^{\left[x,z\right]}\Delta_{c_{x}}^{k}\varsigma_{k}\\ \vdots \\ C_{c,k}^{\left[x,z\right]}{\left(\Delta_{c_{x}}^{k}\right)}^{M_{1}}\varsigma_{k}\\ C_{c,k}^{\left[y,z\right]}\Delta_{c_{y}}^{k}\varsigma_{k}\\ \vdots \\ C_{c,k}^{\left[y,z\right]}{\left(\Delta_{c_{y}}^{k}\right)}^{M_{2}}\varsigma_{k} \end{array}\right]$$

where $T_{c,k}^{-1}$ denotes the coherent eigenvector of the *k*th coherent group, $C_{c,k}^{\left[x,z\right]}=[c_{x,K_{u}+k,1},\cdots ,c_{x,K_{u}+k,p_{k}}]$ and $C_{c,k}^{\left[y,z\right]}=\left[c_{y,K_{u}+k,1},\cdots ,c_{y,K_{u}+k,p_{k}}\right]$. Using the similar way as for Equation (20), ${\hat{A}}_{c,k}$ can be partitioned into four submatrices ${\hat{A}}_{c,k}={\left[{\left({\hat{A}}_{c,k}^{\left[x\right]}\right)}^{T},{\left({\hat{A}}_{c,k}^{\left[z_{x}\right]}\right)}^{T},{\left({\hat{A}}_{c,k}^{\left[y\right]}\right)}^{T},{\left(\;{\hat{A}}_{c,k}^{\left[z_{y}\right]}\right)}^{T}\right]}^{T}$, where ${\hat{A}}_{c,k}^{\left[x\right]}=G_{2M_{1},1}^{T}{\hat{A}}_{c,k}^{\left[x,z\right]}$, ${\hat{A}}_{c,k}^{\left[z_{x}\right]}=G_{2M_{1},2}^{T}{\hat{A}}_{c,k}^{\left[x,z\right]}$, ${\hat{A}}_{c,k}^{\left[y\right]}=G_{2M_{2},1}^{T}{\hat{A}}_{c,k}^{\left[y,z\right]}$, and ${\hat{A}}_{c,k}^{\left[z_{y}\right]}=G_{2M_{2},2}^{T}{\hat{A}}_{c,k}^{\left[y,z\right]}\;$ with ${\hat{A}}_{c,k}^{\left[x,z\right]}$ and ${\hat{A}}_{c,k}^{\left[y,z\right]}$ being the first $2M_{1}$ and the last $2M_{2}$ rows of ${\hat{A}}_{c,k}$. However, ${\hat{A}}_{c,k}$ and its submatrices cannot be applied to DOA estimation directly owing to the rank deficiency (*i.e.*, $\mathrm{rank}\left({\hat{A}}_{c,k}\right)=1$).

Thus, four Hankel matrices are constructed for the purpose of “decorrelating”, that is

(46) $$B_{c,k}^{\left[x\right]}=\left[\begin{array}{cccc} {\hat{A}}_{c,k}^{\left[x\right]}(1) & {\hat{A}}_{c,k}^{\left[x\right]}(2) & \cdots & {\hat{A}}_{c,k}^{\left[x\right]}(p_{k})\\ {\hat{A}}_{c,k}^{\left[x\right]}(2) & {\hat{A}}_{c,k}^{\left[x\right]}(3) & \cdots & {\hat{A}}_{c,k}^{\left[x\right]}(p_{k}+1)\\ \vdots & \vdots & \ddots & \vdots \\ {\hat{A}}_{c,k}^{\left[x\right]}(M_{1}-p_{k}) & {\hat{A}}_{c,k}^{\left[x\right]}(M_{1}-p_{k}+1) & \cdots & {\hat{A}}_{c,k}^{\left[x\right]}(M_{1}) \end{array}\right]$$

(47) $$B_{c,k}^{\left[z_{x}\right]}=\left[\begin{array}{cccc} {\hat{A}}_{c,k}^{\left[z_{x}\right]}(1) & {\hat{A}}_{c,k}^{\left[z_{x}\right]}(2) & \cdots & {\hat{A}}_{c,k}^{\left[z_{x}\right]}(p_{k})\\ {\hat{A}}_{c,k}^{\left[z_{x}\right]}(2) & {\hat{A}}_{c,k}^{\left[z_{x}\right]}(3) & \cdots & {\hat{A}}_{c,k}^{\left[z_{x}\right]}(p_{k}+1)\\ \vdots & \vdots & \ddots & \vdots \\ {\hat{A}}_{c,k}^{\left[z_{x}\right]}(M_{1}-p_{k}) & {\hat{A}}_{c,k}^{\left[z_{x}\right]}(M_{1}-p_{k}+1) & \cdots & {\hat{A}}_{c,k}^{\left[z_{x}\right]}(M_{1}) \end{array}\right]$$

(48) $$B_{c,k}^{\left[y\right]}=\left[\begin{array}{cccc} {\hat{A}}_{c,k}^{\left[y\right]}(1) & {\hat{A}}_{c,k}^{\left[y\right]}(2) & \cdots & {\hat{A}}_{c,k}^{\left[y\right]}(p_{k})\\ {\hat{A}}_{c,k}^{\left[y\right]}(2) & {\hat{A}}_{c,k}^{\left[y\right]}(3) & \cdots & {\hat{A}}_{c,k}^{\left[y\right]}(p_{k}+1)\\ \vdots & \vdots & \ddots & \vdots \\ {\hat{A}}_{c,k}^{\left[y\right]}(M_{2}-p_{k}) & {\hat{A}}_{c,k}^{\left[y\right]}(M_{2}-p_{k}+1) & \cdots & {\hat{A}}_{c,k}^{\left[y\right]}(M_{2}) \end{array}\right]$$

(49) $$B_{c,k}^{\left[z_{y}\right]}=\left[\begin{array}{cccc} {\hat{A}}_{c,k}^{\left[z_{y}\right]}(1) & {\hat{A}}_{c,k}^{\left[z_{y}\right]}(2) & \cdots & {\hat{A}}_{c,k}^{\left[z_{y}\right]}(p_{k})\\ {\hat{A}}_{c,k}^{\left[z_{y}\right]}(2) & {\hat{A}}_{c,k}^{\left[z_{y}\right]}(3) & \cdots & {\hat{A}}_{c,k}^{\left[z_{y}\right]}(p_{k}+1)\\ \vdots & \vdots & \ddots & \vdots \\ {\hat{A}}_{c,k}^{\left[z_{y}\right]}(M_{2}-p_{k}) & {\hat{A}}_{c,k}^{\left[z_{y}\right]}(M_{2}-p_{k}+1) & \cdots & {\hat{A}}_{c,k}^{\left[z_{y}\right]}(M_{2}) \end{array}\right]$$

where $M_{1}-p_{k}>p_{k}$ and $M_{2}-p_{k}>p_{k}$ must be satisfied. It is easy to be proved that the matrices $B_{c,k}^{\left[x\right]}$, $B_{c,k}^{\left[z_{x}\right]}$, $B_{c,k}^{\left[y\right]}$ and $B_{c,k}^{\left[z_{y}\right]}$ are of rank $p_{k}$, which is a precondition for estimating the DOA and polarization parameters of the coherent sources correctly. Therefore, $B_{c,k}^{\left[x\right]}$, $B_{c,k}^{\left[z_{x}\right]}$, $B_{c,k}^{\left[y\right]}$, and $B_{c,k}^{\left[z_{y}\right]}$ can be used in place of ${\hat{A}}_{c,k}^{\left[x\right]}$, ${\hat{A}}_{c,k}^{\left[z_{x}\right]}$, ${\hat{A}}_{c,k}^{\left[y\right]}$, and ${\hat{A}}_{c,k}^{\left[z_{y}\right]}$ that defined in Equations (21)–(24) respectively for the derivation of coherent coarse estimates and fine estimates with cyclical ambiguity, which is similar to what we did for the uncorrelated sources, and hence it is omitted here. In addition, it should be noted that the $p_{k}$ direction-cosines along *x*-axis and the $p_{k}$ direction-cosines along *y*-axis with respect to the *k*th coherent group must obey one-to-one relationship, thus we resort to a simple pair matching method outlined in [8].

The main steps of the proposed method are summarized in Table 2.

## 4. Discussion

To describe the proposed method more comprehensively, several individual properties, computational complexity analysis, and the extension of the proposed method are discussed in this section.

### 4.1. Individual Properties

Unlike the existing DOA estimation methods for a mixture of uncorrelated and coherent sources with vector sensor array, such as the PAS method [13] and the IPAS method [14], the proposed method has some individual properties that should be highlighted.

(1). Estimation of both DOA and polarization parameters. Different from the PAS and the IPAS methods, the proposed method can provide not only the DOA estimates, but also the polarization estimates which can be further utilized for target classification and recognition.
(2). Extended array aperture. The proposed method extends the effective array aperture from two aspects: (1) separating the uncorrelated sources from the coherent sources; (2) extending the inter-sensor spacings beyond a half-wavelength. By contrast, the PAS and IPAS methods are restricted to the spatial Nyquist sampling theorem, that is, the inter-sensor spacing must be no more than a half-wavelength. Thus, the proposed method has a comparatively extended array aperture which enhances the estimation accuracy accordingly.
(3). Reduction in mutual coupling effects and antenna hardware costs. Compared with the spatially collocated six-component vector sensor array used in the existing methods, the number of collocated antennas of the proposed L-shaped SD-VS array is reduced from six to two, which significantly reduces the mutual coupling effects. In addition, the antenna hardware costs are reduced.
(4). Adaptation to SD-VS array with different antenna compositions. The proposed method is applicable to four different antenna compositions as shown in Table 1, not limited to a unique antenna composition, which makes it more suitable for the practical situations.

### 4.2. Computational Complexity

To demonstrate the computational efficiency of the proposed method, we discuss the computational complexities of the proposed method, PAS method, and IPAS method. Note that a large portion of the computational burden is occupied by the multiplication operations as compared to the addition operations, thus here we only consider the multiplication operations during the discussion of the computational complexities.

Table 3 presents the comparison of computational complexity of the three methods, wherein the main computational burden such as the calculation of covariance matrix, EVD, or singular value decomposition (SVD), Moore-Penrose and peak search are considered. L denotes the snapshot number. $\Delta_{s}$ denotes the number of spectral points of the total angular domain, which can be determined by $\Delta_{s}=\Delta_{\theta }\Delta_{\phi }$ with $\Delta_{\theta }$ and $\Delta_{\phi }$ being the sample points of elevation and azimuth angles, respectively.

As can be seen from Table 3, the total computational complexities of the three methods are approximately given by (For convenience comparison, $M_{1}=0.5M$)

(50) $$C_{Pro}\approx M^{3}+M^{2}L+4M\sum_{k=1}^{D}p_{k}^{2}+{\left(K_{u}+D\right)}^{2}M$$

(51) $$C_{PAS}\approx M^{3}+6M^{2}L+\Delta_{s}\left(M^{2}-MK\right)$$

(52) $$\begin{array}{l} C_{IPAS}\approx 1.875M^{3}+M^{2}L+2M\sum_{k=1}^{D}p_{k}^{2}+2{\left(K_{u}+D\right)}^{2}M\\ =0.875M^{3}-2M\sum_{k=1}^{D}p_{k}^{2}+{\left(K_{u}+D\right)}^{2}M+C_{Pro} \end{array}$$

It is seen that the proposed method and the IPAS method have the similar computational complexity, which is much lower than that of the PAS method. Note that the PAS method involves intensive 2-D spectral search operation, in which the inequality $\Delta_{s}\gg M$ holds, hence it requires more computational burdens than the proposed method and the IPAS method. Furthermore, the advantage of the proposed method in terms of computational complexity becomes increasingly obvious with the increase of M and $\Delta_{s}$.

### 4.3. Extension to the Coexistence of Correlated and Coherent Sources

The proposed method can be extended to the scenario where correlated and coherent sources coexist. In such a scenario, the array output vector defined in Equation (4) can be rewritten as

(53) $$x\left(t\right)=\sum_{k=1}^{K_{pc}}a\left(\theta_{k},\phi_{k},\gamma_{k},\eta_{k}\right)s_{k}\left(t\right)+\sum_{k=K_{u}+1}^{K_{u}+D}\;\sum_{p=1}^{p_{k}}a\left(\theta_{k,p},\phi_{k,p},\gamma_{k,p},\eta_{k,p}\right)\varsigma_{k,p}s_{k}\left(t\right)+n\left(t\right)$$

where $K_{pc}$ denotes the number of partially correlated sources. The partially correlated sources can be distinguished from the coherent sources based on the moduli of the eigenvalues, which is similar to the separation method used for a mixture of uncorrelated and coherent sources. Afterwards, the partially correlated sources and the remaining coherent sources are resolved in accordance with the methods in Section 3.2 and Section 3.3, respectively.

## 5. Simulation

In this section, several simulations are presented to illustrate the performance of the proposed method. Consider an L-shaped SD-VS array which contains a total of M=(5+5)×2 dipoles and/or loops, *i.e.*, 20 dipoles and/or loops all altogether. For the sake of convenience, we consider Δx=Δy and $M_{1}=M_{2}$. Two hundred independent Monte Carlo trials are conducted for the following simulations, and the root mean squared error (RMSE) is chosen as a performance metric, which is defined as

(54) $$RMSE=\sqrt{\frac{1}{200\tilde{K}}\sum_{i=1}^{200}\sum_{k=1}^{\tilde{K}}{\left({\hat{\theta }}_{k}-\theta_{k}\right)}^{2}+{\left({\hat{\phi }}_{k}-\phi_{k}\right)}^{2}}$$

where ${\hat{\theta }}_{k}$ and ${\hat{\phi }}_{k}$ are the estimates of $\theta_{k}$ and $\phi_{k}$ in the *k*th Monte Carlo trial, $\tilde{K}$ denotes the number of uncorrelated or coherent sources.

**Figure 2:**
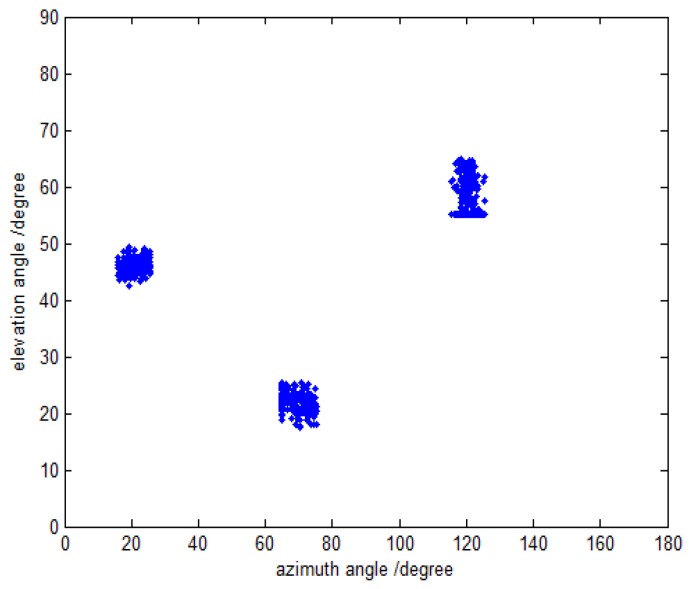
The coarse DOA estimation with fixed SNR 15 dB and snapshot number 500.

**Figure 3:**
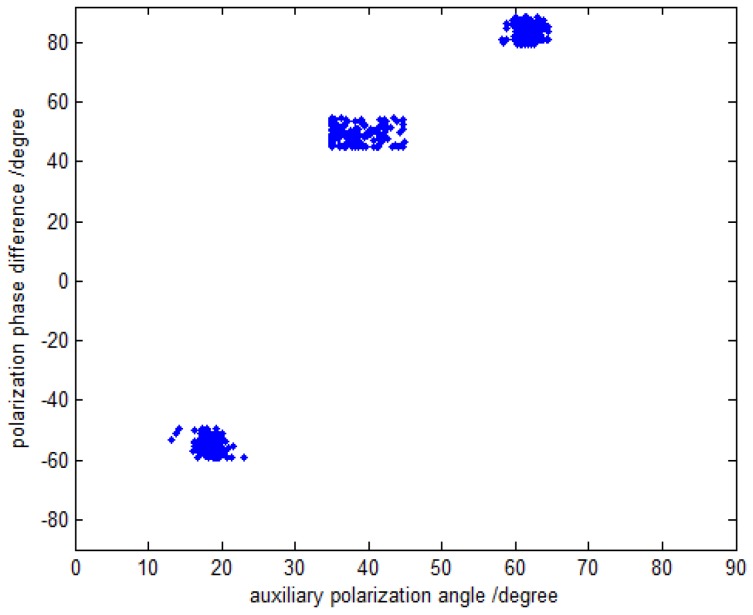
The polarization estimation with fixed SNR 15 dB and snapshot number 500.

**Figure 4:**
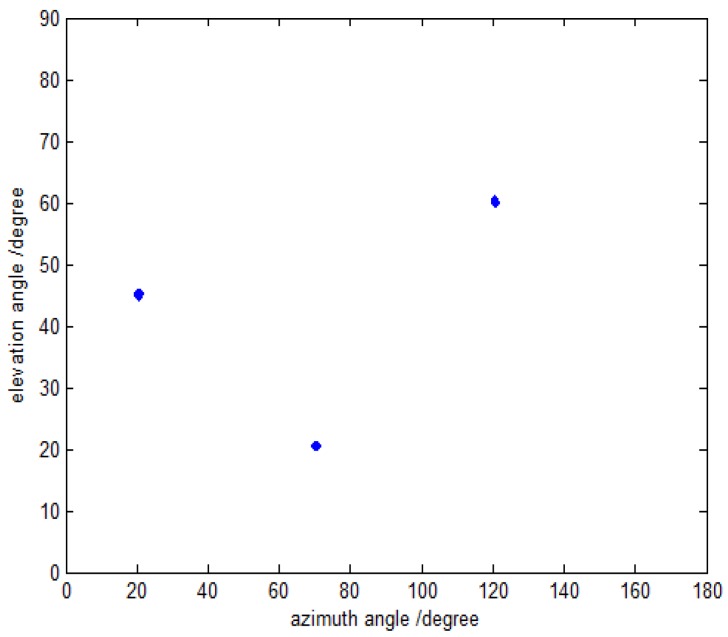
The refined DOA estimation with fixed SNR 15 dB and snapshot number 500.

In the first simulation, we evaluate the DOA and polarization estimation performance of the proposed method. Assume that the three far-field narrowband completely polarized electromagnetic wave sources are composed of one uncorrelated source and two coherent sources impinge on this array. The uncorrelated source is parameterized by $\left\{20.5^{\circ },70.3^{\circ },40^{\circ },50^{\circ }\right\}$, and the coherent sources are parameterized by $\left\{60.0^{\circ },120.6^{\circ },18^{\circ },-54^{\circ }\right\}$ and $\left\{45.2^{\circ },20.7^{\circ },62^{\circ },84^{\circ }\right\}$ with the fading coefficients [1, −0.5280+0.6010j]. The SNR, snapshot number and the inter-sensor spacings are set to be 15 dB, 500 and Δx=Δy=3λ, respectively. The coarse estimates of azimuth-elevation angles, the estimates of polarization parameters and the refined estimates of azimuth-elevation angles are shown in Figure 2, Figure 3 and Figure 4, respectively. It can be seen from Figure 2, Figure 3 and Figure 4 that the proposed method is able to estimate the DOA and polarization parameters of impinging sources efficiently, and the accuracy of the refined estimation has been improved significantly as compared to that of the coarse estimates. The polarization parameters are obtained from the coarse estimates of azimuth-elevation angles, and hence they share the same estimation accuracy with the coarse estimates ofazimuth-elevation angles.

**Figure 5:**
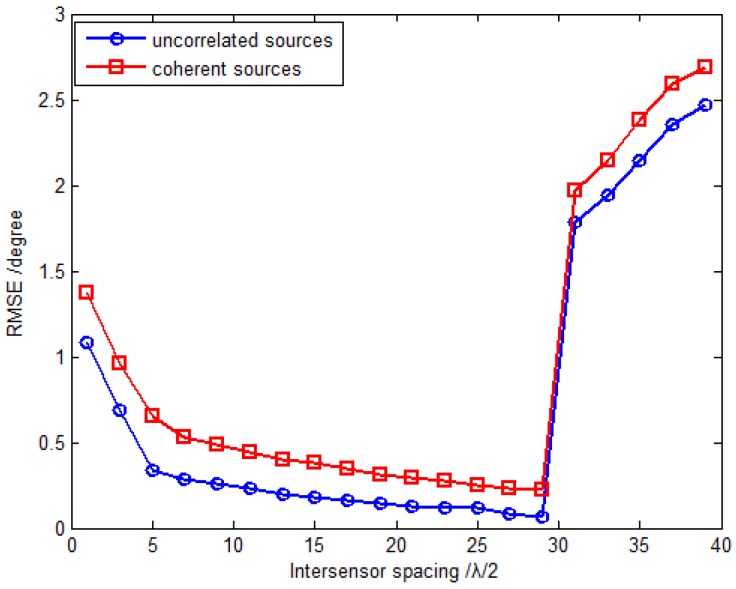
RMSE *versus* inter-sensor spacing with fixed SNR 15 dB and snapshot number 500.

In the second simulation, the RMSE of the proposed method *versus* the inter-sensor spacings is investigated. The simulation settings are the same as those of the first simulation, except that the inter-sensor spacings in this simulation are ranged from 0.5λ to 20λ. Figure 5 plots the RMSE *versus* the inter-sensor spacings with the fixed SNR 15 dB and snapshot number 500. It can be seen from Figure 5 that the RMSE tends to decrease with the increase of inter-sensor spacings under the condition that Δx=Δy≤14.5λ, which is consistent with the foregoing theoretical analysis. However, when further increasing the inter-sensor spacings to Δx=Δy>14.5λ, the RMSEs of both uncorrelated and coherent sources begin to increase. The reason for this phenomenon is that the increase of the inter-sensor spacings means the extension of array aperture, which helps to enhance the estimation accuracy. On the other hand, as the inter-sensor spacings increase, the grid sizes for fine estimates tend to decrease, but the coarse estimates remain unchanged. This implies that the probability that the coarse estimates may identify the wrong grid point will increase accordingly, thus the corresponding estimation performance would degrade seriously.

**Figure 6:**
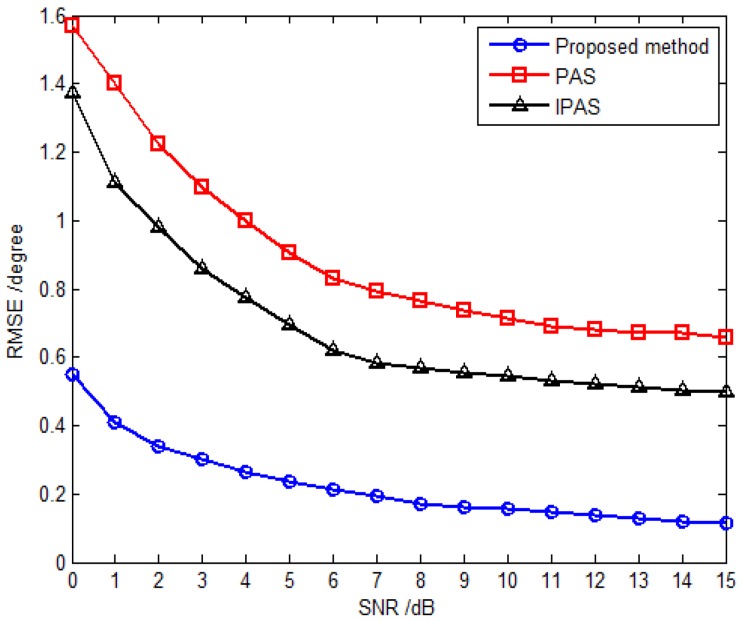
RMSE *versus* SNR for uncorrelated sources with fixed snapshot number 500.

**Figure 7:**
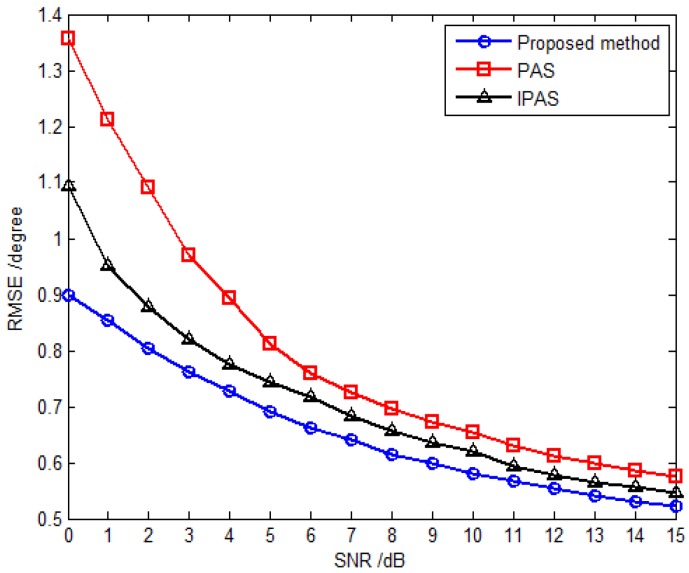
RMSE *versus* SNR for coherent sources with fixed snapshot number 500.

**Figure 8:**
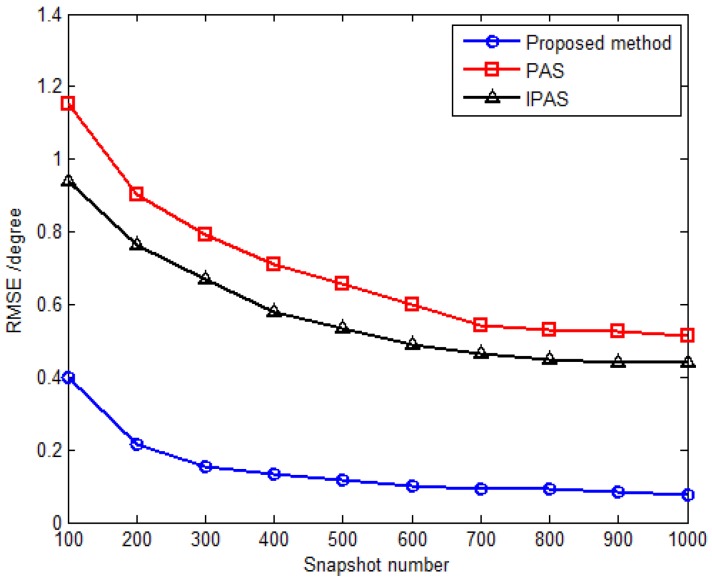
RMSE *versus* snapshot number for uncorrelated sources with fixed SNR 15 dB.

**Figure 9:**
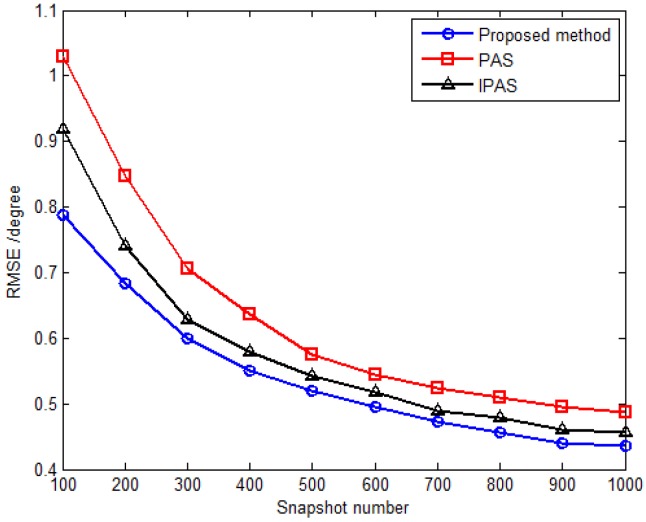
RMSE *versus* snapshot number for coherent sources with fixed SNR 15 dB.

The third simulation compares estimation performance of the proposed method with that of the PAS and IPAS methods *versus* SNR and the snapshot number. For comparison purposes, an 11-element L-shaped six-component vector sensor array is adopted, which contains a total of 11×6 dipoles or loops. Obviously, the antenna hardware cost of the SD-VS array required by the proposed method is significantly less than that of six-component vector sensor array required by the PAS and IPAS methods. Considering one uncorrelated source parameterized by $\left\{30.5^{\circ },69.7^{\circ },30^{\circ },53^{\circ }\right\}$ and two coherent sources are parameterized by $\left\{53.6^{\circ },108.6^{\circ },18^{\circ },-54^{\circ }\right\}$ and $\left\{41.2^{\circ },16.5^{\circ },61^{\circ },79^{\circ }\right\}$ with the fading coefficients [1, −0.3358−0.7261j]. The inter-sensor spacings along the *x*-axis and *y*-axis are set to be Δx=Δy=3λ for the proposed method, while those are set to be Δx=Δy=0.5λ for the PAS and IPAS methods in accordance with the spatial Nyquist sampling theorem. The RMSE *versus* SNR with fixed snapshot number of 500 for uncorrelated and coherent sources are presented in Figure 6 and Figure 7, respectively, while Figure 8 and Figure 9 present the RMSE *versus* snapshot number with fixed SNR of 15 dB for uncorrelated and coherent sources.

The results from Figure 6, Figure 7, Figure 8 and Figure 9 demonstrate that the proposed method yields more accurate DOA estimates than the PAS and IPAS methods. The reason is that both the PAS and the IPAS methods are restricted to the spatial Nyquist sampling theorem, while the proposed method can extend the inter-sensor spacing beyond a half-wavelength. That is to say, the proposed method has a larger array aperture as compared to the PAS and IPAS methods, and hence the estimation accuracy is improved accordingly. Moreover, the PAS method deals with the uncorrelated and coherent sources simultaneously, which leads to a low utilization of the array aperture, while the proposed method and the IPAS method estimate the uncorrelated and coherent sources separately. It should also be noted that the modulus property principle is exploited to eliminate the uncorrelated sources in the proposed method, which causes no power loss of coherent sources. However, the power loss of coherent sources may occur in the IPAS method due to the spatial differencing theory.

**Figure 10:**
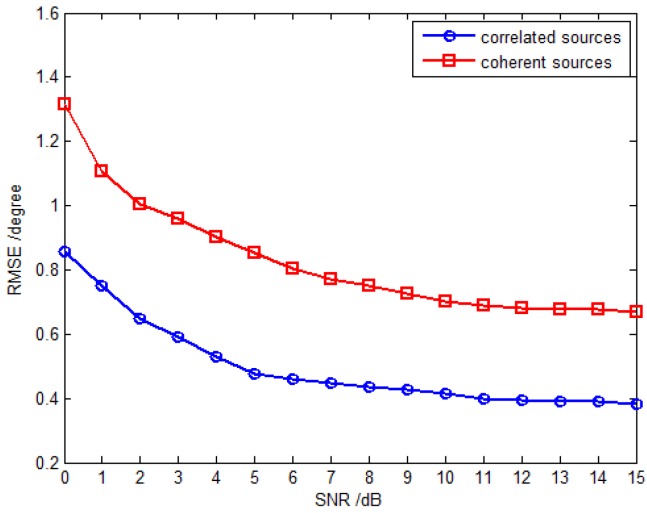
RMSE *versus* SNR with fixed snapshot number 500.

**Figure 11:**
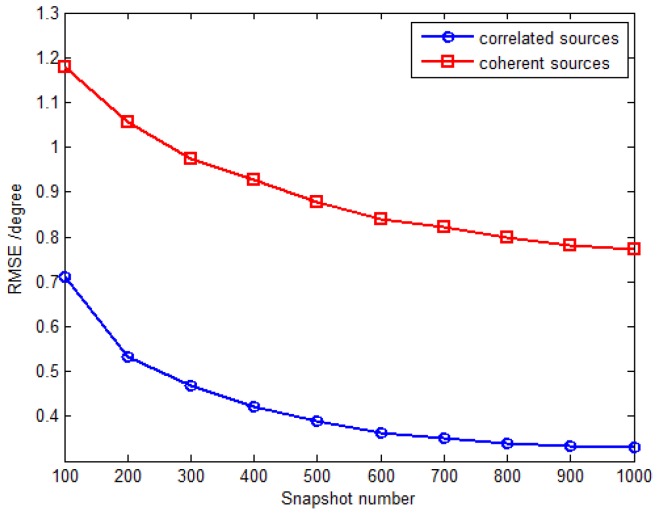
RMSE *versus* snapshot number with fixed SNR 15 dB.

In the last simulation, the proposed method is extended to the scenario where partially correlated and coherent sources coexist. There are two partially correlated sources parameterized by $\left\{32.2^{\circ },40.3^{\circ },34^{\circ },67^{\circ }\right\}$ and $\left\{45.9^{\circ },54.7^{\circ },40^{\circ },-15^{\circ }\right\}$ with the correlation coefficient $\rho e^{j\alpha }=0.3e^{j117.93^{\circ }}$ and two coherent sources parameterized by $\left\{82.7^{\circ },30.6^{\circ },19^{\circ },-45^{\circ }\right\}$ and $\left\{21.2^{\circ },66.7^{\circ },31^{\circ },74^{\circ }\right\}$ with the fading coefficients [1, 0.2891−0.7567j]. The inter-sensor spacings along the *x*-axis and *y*-axis are set to be Δx=Δy=3λ, and the RMSE of DOA estimates for partially correlated sources are defined in a similar way as for the uncorrelated and coherent sources (Equation (53)). Figure 10 shows the RMSE *versus* SNR with the fixed snapshot number of 500, and Figure 11 plots the RMSE *versus* snapshot number with the fixed SNR of 15 dB. The results from Figure 10 and Figure 11 illustrate that the proposed method can be extended to deal with the coexistence of correlated and coherent sources.

## 6. Conclusions

In this paper, we develop an L-shaped sparsely-distributed vector sensor (SD-VS) array with four different antenna compositions, with which a novel 2-D DOA and polarization estimation method is developed for a mixture of uncorrelated and coherent sources. On the basis of the moduli of the eigenvalues, the uncorrelated sources are separated from the coherent sources. Subsequently, the coarse estimates of uncorrelated sources are achieved, and then used as coarse references for the fine estimates with cyclical ambiguity. Finally, four Hankel matrices are constructed for the purpose of “decorrelating”, with which the coherent sources are resolved in a similar way as for the uncorrelated sources. For the proposed L-shaped SD-VS array, the number of collocated antennas of each sensor is two and the inter-sensor spacings can be far larger than a half-wavelength, which reduces the mutual coupling effects and meanwhile extends the array aperture. Moreover, the proposed method has a low computational burden. Simulation results show that the proposed method can estimate both the DOA and polarization parameters of the mixed sources efficiently and has better estimation performance than the PAS and IPAS methods in terms of estimation accuracy.

## Figures and Tables

**Table 1:** Coarse estimates of uncorrelated sources for four different antenna compositions.

Composition	Estimation Formulas	Intermediate Variables
(a)	${\hat{\phi }}_{k}^{coarse}=\left\{ \begin{array}{l} \tan^{-1}\left\{\frac{-\text{Im}\left({\hat{e}}_{u,k}^{\left[x\right]}/{\hat{e}}_{u,k}^{\left[z\right]}\right)}{\text{Im}\left({\hat{e}}_{u,k}^{\left[y\right]}/{\hat{e}}_{u,k}^{\left[z\right]}\right)}\right\},\;\mathrm{if}\;\left(\sin\eta_{k}\cdot \text{Im}\left({\hat{e}}_{u,k}^{\left[y\right]}/{\hat{e}}_{u,k}^{\left[z\right]}\right)\geq 0\right)\\ \tan^{-1}\left\{\frac{-\text{Im}\left({\hat{e}}_{u,k}^{\left[x\right]}/{\hat{e}}_{u,k}^{\left[z\right]}\right)}{\text{Im}\left({\hat{e}}_{u,k}^{\left[y\right]}/{\hat{e}}_{u,k}^{\left[z\right]}\right)}\right\}+\pi ,\;\mathrm{if}\;\left(\sin\eta_{k}\cdot \text{Im}\left({\hat{e}}_{u,k}^{\left[y\right]}/{\hat{e}}_{u,k}^{\left[z\right]}\right)<0\right) \end{array}\right.$ ${\hat{\theta }}_{k}^{coarse}=\left\{ \begin{array}{l} \tan^{-1}\left\{\frac{1}{H}\right\},\;\mathrm{if}\;\left(H\geq 0\right)\\ \tan^{-1}\left\{\frac{1}{H}\right\}+\pi ,\;\mathrm{if}\;\left(H<0\right)\; \end{array}\right.$ ${\hat{\eta }}_{k}^{coarse}=-∠\left(\left({\hat{e}}_{u,k}^{\left[x\right]}/{\hat{e}}_{u,k}^{\left[z\right]}\right)\sin{\hat{\phi }}_{k}^{coarse}-\left({\hat{e}}_{u,k}^{\left[y\right]}/{\hat{e}}_{u,k}^{\left[z\right]}\right)\cos{\hat{\phi }}_{k}^{coarse}\right)$ ${\hat{\gamma }}_{k}^{coarse}=\cot^{-1}\left(\frac{\text{Im}\left({\hat{e}}_{u,k}^{\left[y\right]}/{\hat{e}}_{u,k}^{\left[z\right]}\right)\sin{\hat{\phi }}_{k}^{coarse}}{\cos{\hat{\phi }}_{k}^{coarse}\sin{\hat{\eta }}_{k}^{coarse}}\right)$	$\frac{{\hat{e}}_{u,k}^{\left[x\right]}}{{\hat{e}}_{u,k}^{\left[z\right]}}=\left[-\cot\theta_{k}\cos\phi_{k}+\cot\gamma_{k}\frac{\sin\phi_{k}}{\sin\theta_{k}}\cos\eta_{k}\right]+j\left[-\cot\gamma_{k}\frac{\sin\phi_{k}}{\sin\theta_{k}}\sin\eta_{k}\right]$ $\frac{{\hat{e}}_{u,k}^{\left[y\right]}}{{\hat{e}}_{u,k}^{\left[z\right]}}=\left[-\cot\theta_{k}\sin\phi_{k}-\cot\gamma_{k}\frac{\cos\phi_{k}}{\sin\theta_{k}}\cos\eta_{k}\right]+j\left[\cot\gamma_{k}\frac{\cos\phi_{k}}{\sin\theta_{k}}\sin\eta_{k}\right]$ $H=-\text{Re}\left({\hat{e}}_{u,k}^{\left[x\right]}/{\hat{e}}_{u,k}^{\left[z\right]}\right)\cos{\hat{\phi }}_{k}^{coarse}-\text{Re}\left(\left({\hat{e}}_{u,k}^{\left[y\right]}/{\hat{e}}_{u,k}^{\left[z\right]}\right)\right)\sin{\hat{\phi }}_{k}^{coarse}$
(b)	${\hat{\phi }}_{k}^{coarse}=\left\{ \begin{array}{l} \tan^{-1}\left\{\frac{-\text{Im}\left({\hat{h}}_{u,k}^{\left[x\right]}/{\hat{h}}_{u,k}^{\left[z\right]}\right)}{\text{Im}\left({\hat{h}}_{u,k}^{\left[y\right]}/{\hat{h}}_{u,k}^{\left[z\right]}\right)}\right\},\;\mathrm{if}\;\left(\sin\eta_{k}\cdot \text{Im}\left({\hat{h}}_{u,k}^{\left[y\right]}/{\hat{h}}_{u,k}^{\left[z\right]}\right)\geq 0\right)\\ \tan^{-1}\left\{\frac{-\text{Im}\left({\hat{h}}_{u,k}^{\left[x\right]}/{\hat{h}}_{u,k}^{\left[z\right]}\right)}{\text{Im}\left({\hat{h}}_{u,k}^{\left[y\right]}/{\hat{h}}_{u,k}^{\left[z\right]}\right)}\right\}+\pi ,\;\mathrm{if}\;\left(\sin\eta_{k}\cdot \text{Im}\left({\hat{h}}_{u,k}^{\left[y\right]}/{\hat{h}}_{u,k}^{\left[z\right]}\right)<0\right)\; \end{array}\right.$ ${\hat{\theta }}_{k}^{coarse}=\left\{ \begin{array}{l} \tan^{-1}\left\{\frac{1}{H}\right\},\;\mathrm{if}\;\left(H\geq 0\right)\\ \tan^{-1}\left\{\frac{1}{H}\right\}+\pi ,\;\mathrm{if}\;\left(H<0\right)\; \end{array}\right.$ ${\hat{\eta }}_{k}^{coarse}=-∠\left(\left({\hat{h}}_{u,k}^{\left[x\right]}/{\hat{h}}_{u,k}^{\left[z\right]}\right)\cos{\hat{\phi }}_{k}^{coarse}-\left({\hat{h}}_{u,k}^{\left[y\right]}/{\hat{h}}_{u,k}^{\left[z\right]}\right)\sin{\hat{\phi }}_{k}^{coarse}\right)$ ${\hat{\gamma }}_{k}^{coarse}=\tan^{-1}\left(\frac{\text{Im}\left({\hat{h}}_{u,k}^{\left[y\right]}/{\hat{h}}_{u,k}^{\left[z\right]}\right)\sin{\hat{\theta }}_{k}^{coarse}}{\cos{\hat{\phi }}_{k}^{coarse}\sin{\hat{\eta }}_{k}^{coarse}}\right)$	$\frac{{\hat{h}}_{u,k}^{\left[x\right]}}{{\hat{h}}_{u,k}^{\left[z\right]}}=\left[-\cot\theta_{k}\cos\phi_{k}-\tan\gamma_{k}\frac{\sin\phi_{k}}{\sin\theta_{k}}\cos\eta_{k}\right]+j\left[-\tan\gamma_{k}\frac{\sin\phi_{k}}{\sin\theta_{k}}\sin\eta_{k}\right]$ $\frac{{\hat{h}}_{u,k}^{\left[y\right]}}{{\hat{h}}_{u,k}^{\left[z\right]}}=\left[-\cot\theta_{k}\sin\phi_{k}+\tan\gamma_{k}\frac{\cos\phi_{k}}{\sin\theta_{k}}\cos\eta_{k}\right]+j\left[\tan\gamma_{k}\frac{\cos\phi_{k}}{\sin\theta_{k}}\sin\eta_{k}\right]$ $H=-\text{Re}\left({\hat{h}}_{u,k}^{\left[x\right]}/{\hat{h}}_{u,k}^{\left[z\right]}\right)\cos{\hat{\phi }}_{k}^{coarse}-\text{Re}\left({\hat{h}}_{u,k}^{\left[y\right]}/{\hat{h}}_{u,k}^{\left[z\right]}\right)\sin{\hat{\phi }}_{k}^{coarse}$
(c)	${\hat{\phi }}_{k}^{coarse}=\left\{ \begin{array}{l} \cot^{-1}\left\{\frac{\text{Im}\left({\hat{h}}_{u,k}^{\left[x\right]}/{\hat{e}}_{u,k}^{\left[z\right]}\right)}{\text{Im}\left({\hat{h}}_{u,k}^{\left[y\right]}/{\hat{e}}_{u,k}^{\left[z\right]}\right)}\right\},\;\mathrm{if}\;\left(\sin\eta_{k}\cdot \text{Im}\left({\hat{h}}_{u,k}^{\left[x\right]}/{\hat{e}}_{u,k}^{\left[z\right]}\right)\geq 0\right)\\ \cot^{-1}\left\{\frac{\text{Im}\left({\hat{h}}_{u,k}^{\left[x\right]}/{\hat{e}}_{u,k}^{\left[z\right]}\right)}{\text{Im}\left({\hat{h}}_{u,k}^{\left[y\right]}/{\hat{e}}_{u,k}^{\left[z\right]}\right)}\right\}+\pi ,\;\mathrm{if}\;\left(\sin\eta_{k}\cdot \text{Im}\left({\hat{h}}_{u,k}^{\left[x\right]}/{\hat{e}}_{u,k}^{\left[z\right]}\right)<0\right)\; \end{array}\right.$ ${\hat{\theta }}_{k}^{coarse}=\left\{ \begin{array}{l} \sin^{-1}\left\{\frac{1}{H}\right\},\;\mathrm{if}\;\theta_{k}\in \left[0,\;\pi /2\right]\\ \pi -\sin^{-1}\left\{\frac{1}{H}\right\},\;\mathrm{if}\;\theta_{k}\in \left(\pi /2,\pi \right]\; \end{array}\right.$ ${\hat{\eta }}_{k}^{coarse}=-∠\left(\left({\hat{h}}_{u,k}^{\left[x\right]}/{\hat{e}}_{u,k}^{\left[z\right]}\right)\cos{\hat{\phi }}_{k}^{coarse}+\left({\hat{h}}_{u,k}^{\left[y\right]}/{\hat{e}}_{u,k}^{\left[z\right]}\right)\sin{\hat{\phi }}_{k}^{coarse}\right)$ ${\hat{\gamma }}_{k}^{coarse}=\cot^{-1}\left(\frac{-\text{Im}\left({\hat{h}}_{u,k}^{\left[y\right]}/{\hat{e}}_{u,k}^{\left[z\right]}\right)}{\sin{\hat{\phi }}_{k}^{coarse}\cot{\hat{\theta }}_{k}^{coarse}\sin{\hat{\eta }}_{k}^{coarse}}\right)$	$\frac{{\hat{h}}_{u,k}^{\left[x\right]}}{{\hat{e}}_{u,k}^{\left[z\right]}}=\left[\frac{\sin\phi_{k}}{\sin\theta_{k}}+\cos\phi_{k}\cot\theta_{k}\cot\gamma_{k}\cos\eta_{k}\right]+j\left[-\cos\phi_{k}\cot\theta_{k}\cot\gamma_{k}\sin\eta_{k}\right]$ $\frac{{\hat{h}}_{u,k}^{\left[y\right]}}{{\hat{e}}_{u,k}^{\left[z\right]}}=\left[-\frac{\cos\phi_{k}}{\sin\theta_{k}}+\sin\phi_{k}\cot\theta_{k}\cot\gamma_{k}\cos\eta_{k}\right]+j\left[-\sin\phi_{k}\cot\theta_{k}\cot\gamma_{k}\sin\eta_{k}\right]$ $H=-\text{Re}\left({\hat{h}}_{u,k}^{\left[x\right]}/{\hat{e}}_{u,k}^{\left[z\right]}\right)\sin{\hat{\phi }}_{k}^{coarse}-\text{Re}\left({\hat{h}}_{u,k}^{\left[y\right]}/{\hat{e}}_{u,k}^{\left[z\right]}\right)\cos{\hat{\phi }}_{k}^{coarse}$
(d)	${\hat{\phi }}_{k}^{coarse}=\left\{ \begin{array}{l} \cot^{-1}\left\{\frac{\text{Im}\left({\hat{e}}_{u,k}^{\left[x\right]}/{\hat{h}}_{u,k}^{\left[z\right]}\right)}{\text{Im}\left({\hat{e}}_{u,k}^{\left[y\right]}/{\hat{h}}_{u,k}^{\left[z\right]}\right)}\right\},\;\mathrm{if}\;\left(\sin\eta_{k}\cdot \text{Im}\left({\hat{e}}_{u,k}^{\left[x\right]}/{\hat{h}}_{u,k}^{\left[z\right]}\right)\geq 0\right)\\ \cot^{-1}\left\{\frac{\text{Im}\left({\hat{e}}_{u,k}^{\left[x\right]}/{\hat{h}}_{u,k}^{\left[z\right]}\right)}{\text{Im}\left({\hat{e}}_{u,k}^{\left[y\right]}/{\hat{h}}_{u,k}^{\left[z\right]}\right)}\right\}+\pi ,\;\mathrm{if}\;\left(\sin\eta_{k}\cdot \text{Im}\left({\hat{e}}_{u,k}^{\left[x\right]}/{\hat{h}}_{u,k}^{\left[z\right]}\right)<0\right) \end{array}\right.$ ${\hat{\theta }}_{k}^{coarse}=\left\{ \begin{array}{l} \sin^{-1}\left\{\frac{1}{H}\right\},\;\mathrm{if}\;\theta_{k}\in \left[0,\;\pi /2\right]\\ \pi -\sin^{-1}\left\{\frac{1}{H}\right\},\;\mathrm{if}\;\theta_{k}\in \left(\pi /2,\pi \right] \end{array}\right.$ ${\hat{\eta }}_{k}^{coarse}=∠\left(\left({\hat{e}}_{u,k}^{\left[x\right]}/{\hat{h}}_{u,k}^{\left[z\right]}\right)\cos{\hat{\phi }}_{k}^{coarse}+\left({\hat{e}}_{u,k}^{\left[y\right]}/{\hat{h}}_{u,k}^{\left[z\right]}\right)\sin{\hat{\phi }}_{k}^{coarse}\right)$ ${\hat{\gamma }}_{k}^{coarse}=\tan^{-1}\left(\frac{\text{Im}\left({\hat{e}}_{u,k}^{\left[x\right]}/{\hat{h}}_{u,k}^{\left[z\right]}\right)}{\sin{\hat{\phi }}_{k}^{coarse}\cot{\hat{\theta }}_{k}^{coarse}\sin{\hat{\eta }}_{k}^{coarse}}\right)$	$\frac{{\hat{e}}_{u,k}^{\left[x\right]}}{{\hat{h}}_{u,k}^{\left[z\right]}}=\left[-\frac{\sin\phi_{k}}{\sin\theta_{k}}+\cos\phi_{k}\cot\theta_{k}\tan\gamma_{k}\cos\eta_{k}\right]+j\left[\cos\phi_{k}\cot\theta_{k}\tan\gamma_{k}\sin\eta_{k}\right]$ $\frac{{\hat{e}}_{u,k}^{\left[y\right]}}{{\hat{h}}_{u,k}^{\left[z\right]}}=\left[\frac{\cos\phi_{k}}{\sin\theta_{k}}+\sin\phi_{k}\cot\theta_{k}\tan\gamma_{k}\cos\eta_{k}\right]+j\left[\sin\phi_{k}\cot\theta_{k}\tan\gamma_{k}\sin\eta_{k}\right]$ $H=-\text{Re}\left({\hat{e}}_{u,k}^{\left[x\right]}/{\hat{h}}_{u,k}^{\left[z\right]}\right)\cos{\hat{\phi }}_{k}^{coarse}-\text{Re}\left({\hat{e}}_{u,k}^{\left[y\right]}/{\hat{h}}_{u,k}^{\left[z\right]}\right)\sin{\hat{\phi }}_{k}^{coarse}$

**Table 2:** The main steps of the proposed method.

**Input**: x(1),x(2),⋯,x(N) 1. Obtain X according to Equation (7)
**Distinguish Uncorrelated Sources from Coherent Sources:** 2. Calculate the covariance matrix R of X via Equation (8) 3. Divide $E_{s}$ into four submatrices according to Equation (10) 4. Calculate ${\left(E_{s}^{{\left[x\right]}_{1}}\right)}^{†}E_{s}^{{\left[x\right]}_{2}}$ according to Equation (18) 5. Distinguish uncorrelated sources from coherent sources based on the moduli of the eigenvalues
**Parameter Estimation for Uncorrelated Sources:** 6. Coarse estimates of DOA and polarization 6-1. Estimate $A_{u}$ via Equation (19) and obtain ${\hat{A}}_{u,k}^{\left[x\right]}$, ${\hat{A}}_{u,k}^{\left[z_{x}\right]}$, ${\hat{A}}_{u,k}^{\left[y\right]}$ and ${\hat{A}}_{u,k}^{\left[z_{y}\right]}$ from Equations (21)–(24) 6-2. Compute ${\hat{e}}_{u,k}^{\left[x\right]}/{\hat{e}}_{u,k}^{\left[z\right]}$ and ${\hat{e}}_{u,k}^{\left[y\right]}/{\hat{e}}_{u,k}^{\left[z\right]}$via Equations (25) and (26) 6-3. The coarse estimates of DOA and polarization are obtained from Equations (27)–(30) and the corresponding direction-cosines along *x*-axis and *y*-axis are acquired. 7. Fine estimates of DOA with cyclical ambiguity 7-1. Estimate the *x*-axis and *y*-axis spatial phase factors via Equations (31) and (32) 7-2. Estimate fine but cyclically ambiguous *x*-axis and *y*-axis direction-cosines using Equations (37)–(40) 8. Disambiguate the fine estimates by using the coarse estimates
**Parameter Estimation for Coherent Sources:** 9. Estimate ${\hat{A}}_{c,k}$ via Equation (45) and then partition it into four submatrices: ${\hat{A}}_{c,k}^{\left[x\right]}$, ${\hat{A}}_{c,k}^{\left[z_{x}\right]}$, ${\hat{A}}_{c,k}^{\left[y\right]}$, and ${\hat{A}}_{c,k}^{\left[z_{y}\right]}$ 10. Construct four Hankel matrices according to Equations (46)–(49) for “decorrelating” 11. For the coherent sources, the coarse estimates and the fine estimates with cyclical ambiguity are obtained by utilizing four Hankel matrices, and then the coarse estimates serve as references for disambiguating the fine estimates.

**Table 3:** Comparison of computational complexity of three methods.

Methods	Covariance Matrix	EVD/SVD	Moore-Penrose	Peak Search
Proposed	$M^{2}L$	$M^{3}+4p_{k}^{3}$	${\left(K_{u}+D\right)}^{3}+2{\left(K_{u}+D\right)}^{2}M_{1}+4p_{k}^{3}+2p_{k}^{2}\left(M-2p_{k}-2\right)+2p_{k}^{2}\left(M-2p_{k}\right)+4M-4$	without
PAS	$6M^{2}L$	$M^{3}$	without	$\Delta_{s}\left(M+1\right)\times \left(M-K\right)$
IPAS	$M^{2}L+6{\left(0.5M\right)}^{3}$	$M^{3}+2{\left(K_{u}+D\right)}^{3}+{\left(0.5M\right)}^{3}+2p_{k}^{3}$	$2\left[{\left(K_{u}+D\right)}^{3}+2{\left(K_{u}+D\right)}^{2}\left(0.5M-1\right)\right]+2\left[p_{k}^{3}+2p_{k}^{2}\left(0.5M-1\right)\right]$	without

## Abbreviations

The following abbreviations are used in this manuscript:

DOA	direction of arrival
2-D	two-dimensional
SD-VS	sparsely-distributed vector sensor
TEM	transverse electromagnetic
RMSE	the root mean squared error
